# Low Body Mass Index but Not Obesity Is Associated With In-Hospital Adverse Events and Mortality Among Implantable Cardioverter-Defibrillator Recipients: Insights From the National Cardiovascular Data Registry

**DOI:** 10.1161/JAHA.112.003863

**Published:** 2012-12-19

**Authors:** Jonathan C. Hsu, Paul D. Varosy, Haikun Bao, Yongfei Wang, Jeptha P. Curtis, Gregory M. Marcus

**Affiliations:** Section of Cardiac Electrophysiology, Division of Cardiology, Department of Medicine, University of California, San Francisco, CA (J.C.H., G.M.M.); VA Eastern Colorado Health Care System, University of Colorado, and the Colorado Cardiovascular Outcomes Research (CCOR) Group, Denver, Colorado (P.D.V.); Section of Cardiovascular Medicine, Department of Internal Medicine, Yale University School of Medicine, New Haven, Connecticut (H.B., Y.W., J.P.C.)

**Keywords:** adverse event complications, body mass index, implantable cardioverter-defibrillator, mortality, national registries

## Abstract

**Background:**

Implantable cardioverter-defibrillators (ICDs) are placed in patients at risk for sudden cardiac death, but the procedure may cause adverse events. Patient body habitus may be an important factor responsible for ICD implantation complications. We assessed whether underweight or obese compared with normal weight patients, as defined by body mass index (BMI), were at increased risk for adverse events from ICD implantation.

**Methods and Results:**

We studied 83 312 first-time ICD recipients in the National Cardiovascular Data Registry-ICD Registry implanted between April 2010 and June 2011. Using hierarchical multivariable logistic regression adjusted for patient demographic and clinical characteristics, we examined the association of BMI with in-hospital complications, length of hospital stay, and mortality. Underweight (BMI ≤18.5 kg/m^2^) patients comprised 1.7% of the cohort (n=1434), whereas obese (BMI≥30 kg/m^2^) patients comprised 40.1% (n=33 339). Overall, a higher proportion of underweight patients experienced complications (normal weight, 2.3%; obese, 2.1%; underweight 5.2%; *P*<0.0001) and death (normal weight, 0.3%; obese, 0.3%; underweight 0.8%; *P*=0.026) as a result of ICD implantation. After multivariable adjustment, underweight ICD recipients had a greater odds of complications (odds ratio [OR], 2.15; 95% confidence interval [CI], 1.68 to 2.75; *P*<0.0001), hospital stay >3 days (OR, 1.62; 95% CI, 1.38 to 1.89; *P*<0.0001), and in-hospital death (OR, 2.27; 95% CI, 1.21 to 4.27; *P*=0.011) compared with normal weight patients. Obese patients did not exhibit any meaningful differences in the same outcomes.

**Conclusions:**

In a large, real-world population, underweight first-time ICD recipients experienced significantly more periprocedural complications, prolonged hospital stays, and in-hospital death compared with normal weight patients.

## Introduction

Underweight patients with cardiovascular disease have been relatively understudied, with attention generally focused on the obesity epidemic in the United States and its association with adverse cardiovascular outcomes such as congestive heart failure.^1,2^ Differences in adverse events experienced by underweight and obese patients undergoing heart failure therapies such as implantation with an implantable cardioverter-defibrillator (ICD) have not been extensively studied but may help identify a subgroup of patients at risk for worse outcomes.

Several randomized controlled trials have shown that treatment with an ICD improves survival in patients with systolic heart failure.^3–5^ ICD implantation requires subcutaneous pocket formation, venous access for transvenous leads, generator device placement, and often induction of ventricular fibrillation for defibrillation testing. Complications from any of these steps may be influenced by patient characteristics, such as body mass index (BMI). Previous studies in patients undergoing open heart surgery have found that a lower BMI predicts surgical complications and mortality.^6–8^ However, it is not known whether underweight status or obesity is associated with adverse outcomes in patients with cardiovascular disease undergoing a lower-risk procedure, such as ICD implantation.

We analyzed data from the ICD Registry of the National Cardiovascular Data Registry (NCDR), a national registry of ICD implantations that captures detailed clinical patient information and in-hospital outcomes. By assessing a large population of ICD recipients, we sought to examine whether underweight status and obesity, as defined by BMI, were associated with the risk of procedural complications, length of hospital stay, and risk of in-hospital death.

## Methods

### Data Source

The NCDR ICD registry was created in 2006 to meet the requirements of the Centers for Medicare & Medicaid Services Coverage with Evidence Development decision.^9^ The Heart Rhythm Society and American College of Cardiology collaborated to establish a national registry of ICD implantations, funded by a combination of hospital fees and grants from payers and device companies. Hospitals are mandated to provide data on Medicare beneficiaries receiving an ICD for primary prevention of sudden cardiac death; however, 71.5% of the 1375 participating hospitals are providing data on all patients undergoing ICD implantation. These hospitals submit 88.4% of all ICD implants included in the registry.^9^ This study was limited to patients enrolled after the April 2010 implementation of Version 2.0 of the NCDR ICD Registry, which included BMI data.

### Study Population

After passing its data quality standards, all patients with implant data submitted to the registry between April 1, 2010, and June 30, 2011, were considered for analysis (n=186 307). Patients who received an implant at a hospital that did not report all device implantations (n=13 847), those with a previous pacemaker or ICD (n=85 235), those with an epicardial lead placed during the index procedure (n=2696), those missing data on intended ICD versus cardiac resynchronization therapy with defibrillator implantation (n=31), and those with inaccurate (BMI <10 kg/m^2^ or >60 kg/m^2^) or missing BMI data (n=1186) were excluded from the analysis, leaving 83 312 patients.

### Definition of Underweight, Normal Weight, and Obese

Body habitus was categorized based on World Health Organization classification of BMI values.^10,11^ BMI was calculated by dividing weight in kilograms by the square of height in meters (kg/m^2^) as a simple index of weight-for-height. Underweight and obese patients were defined as a BMI ≤18.5 kg/m^2^ and BMI ≥30 kg/m^2^, respectively.

### Adverse Outcomes

The first outcome was the occurrence of any in-hospital complication during or after ICD implantation. Complications were further categorized as major or minor. Major complications included lead dislodgement, pneumothorax, cardiac arrest, coronary venous dissection, pericardial tamponade, device-related infection, cardiac perforation, transient ischemic attack or stroke, myocardial infarction, urgent cardiac surgery, hemothorax, peripheral embolus, and valve injury. Minor complications included hematoma, drug reaction, conduction block, set screw problem, venous obstruction, and peripheral nerve injury. Patients were categorized as having a major complication if at least one major complication occurred, whereas patients were categorized as having a minor complication if at least one minor complication without a major complication occurred. The second outcome was length of hospital stay quantified by total days spent in the hospital, from implantation to discharge. For analytical purposes, length of hospital stay was dichotomized to >3 days or ≤3 days based on the distribution of the cohort. The third outcome was the occurrence of in-hospital death during or after ICD implantation.

### Statistical Analysis

Normally distributed continuous variables are expressed as mean and SD values, and continuous variables with skewed distributions are expressed as medians and interquartile ranges (IQR). Continuous variables across multiple categories were analyzed by ANOVA or the Kruskal–Wallis test, as appropriate. Categorical variables were compared using the χ^2^ test. Hierarchical logistic regression models were developed to assess the independent association of BMI and each outcome of interest by accounting for regional differences in demographics and clustering of patients within hospitals.^12^ Random effects were used to model the heterogeneity between hospitals. Clustering of patients within hospitals was addressed by including random hospital-specific intercepts in the hierarchical models. Covariates adjusted for in the multivariable models included demographics (age, sex, race), comorbidities (congestive heart failure, New York Heart Association class, syncope, ventricular tachycardia, atrial fibrillation, nonischemic cardiomyopathy, ischemic heart disease, previous myocardial infarction, previous coronary artery bypass graft surgery, previous percutaneous coronary intervention, cerebrovascular disease, chronic lung disease, diabetes, sleep apnea, hypertension, end-stage renal disease, cardiac arrest), and diagnostic information (left ventricular ejection fraction, QRS duration, ECG conduction abnormality, blood urea nitrogen level, serum creatinine).

The prevalence of missing values was very low for all variables (<1%), except for left ventricular ejection fraction (1.65%), blood urea nitrogen level (1.18%), and QRS duration (1.25%). Missing values were imputed to avoid casewise deletion. For categorical variables with a low rate of missing values, the most common response was imputed. For continuous variables, missing values were imputed with the median value among those with data available and dummy variables were created to indicate where the variable was missing. Both imputed data and dummy variables were included in all multivariable models. We repeated the analysis using multiple imputation to handle missing values, which produced no meaningful changes of the results. Statistical tests were 2-sided and considered significant if they yielded a value of *P*<0.05. Analyses were performed using the SAS Statistical Package version 9.2 (SAS Institute, Cary, NC).

## Results

The majority of ICD recipients were normal weight (58.3%, n=48 539), although a large proportion of patients were obese (40.1%, n=33 339), followed by underweight patients (1.7%, n=1434). The median BMI in the obese was 34.3 kg/m^2^ (IQR, 31.8 to 38.4), in the normal weight was 25.7 kg/m^2^ (IQR, 23.4 to 27.8), and in the underweight was 17.4 kg/m^2^ (IQR, 16.4 to 18.1). Baseline characteristics of ICD recipients by BMI category are presented in Table 1. Underweight and obese patients were younger and less commonly white compared with normal weight patients. Women were more commonly underweight. The payer for underweight patients was more often Medicare/Medicaid and less often private insurance. There were several other statistically significant differences in patient demographic characteristics and comorbid conditions across BMI categories (Table 1). Patients who had accurately recorded measurements of BMI differed from the 1186 patients who did not have this data available with respect to several baseline characteristics (Table 2). During ICD implantation procedures, there were differences in the proportion of patients induced for defibrillation testing across BMI categories (73.5% in the obese versus 72.6% in the normal weight versus 69.8% in the underweight; *P*=0.0005).

**Table 1.** Baseline Characteristics of ICD Recipients Stratified by BMI

Characteristic	Underweight, BMI ≤18.5 kg/m^2^ (n=1434)	Normal Weight, 18.5 kg/m^2^<BMI<30 kg/m^2^ (n=48 539)	Obese, BMI ≥30 kg/m^2^ (n=33 339)	*P*
Patient demographic characteristics				

Age, mean (SD), y	65.0 (16.8)	66.4 (13.5)	61.4 (12.7)	<0.0001

Male sex	773 (53.9)	35 638 (73.4)	23 297 (69.9)	<0.0001

Race				<0.0001

White	1125 (78.5)	40 353 (83.1)	26 641 (79.9)	
	
Black	251 (17.5)	6546 (13.5)	5982 (17.9)	
	
Asian	40 (2.8)	1004 (2.1)	224 (0.7)	
	
Other	18 (1.3)	636 (1.3)	492 (1.5)	

Hispanic ethnicity	63 (4.4)	2910 (6.0)	1868 (5.6)	0.004

Insurance payer				

Private	739 (51.5)	29 264 (60.3)	20 206 (60.6)	<0.0001

Medicare	902 (62.9)	29 174 (60.1)	16 450 (49.3)	<0.0001

Medicaid	253 (17.6)	5649 (11.6)	4723 (14.2)	<0.0001

State specific (non-Medicaid)	23 (1.6)	780 (1.6)	605 (1.8)	<0.0001

Other or self-pay	101 (7.0)	3413 (7.0)	2473 (7.4)	0.107

Patient history and risk factors				

Congestive heart failure	1127 (78.6)	36 731 (75.7)	26 162 (78.5)	<0.0001

NYHA class				<0.0001

I	220 (15.3)	8031 (16.5)	4793 (14.4)	
	
II	409 (28.5)	17 391 (35.8)	11 735 (35.2)	
	
III	739 (51.5)	21 533 (44.4)	15 831 (47.5)	
	
IV	66 (4.6)	1584 (3.3)	980 (2.9)	

Nonischemic dilated cardiomyopathy	580 (40.4)	15 887 (32.7)	13 198 (39.6)	<0.0001

Ischemic heart disease	737 (51.4)	30 360 (62.5)	18 704 (56.1)	<0.0001

Previous myocardial infarction	608 (42.4)	24 940 (51.4)	15 431 (46.3)	<0.0001

Previous CABG	302 (21.1)	14 956 (30.8)	8851 (26.5)	<0.0001

Previous PCI	369 (25.7)	16 075 (33.1)	10 514 (31.5)	<0.0001

Syncope	289 (20.2)	8613 (17.7)	5303 (15.9)	<0.0001

Ventricular tachycardia	465 (32.4)	15 755 (32.5)	10 405 (31.2)	0.0008

Cardiac arrest	188 (13.1)	5761 (11.9)	3536 (10.6)	<0.0001

Atrial fibrillation/atrial flutter	381 (26.6)	13 627 (28.1)	9458 (28.4)	0.261

Hypertension	950 (66.2)	36 627 (75.5)	27 778 (83.3)	<0.0001

Diabetes	271 (18.9)	15 308 (31.5)	16 199 (48.6)	<0.0001

Cerebrovascular disease	247 (17.2)	7655 (15.8)	4386 (13.2)	<0.0001

End-stage renal disease	62 (4.3)	1713 (3.5)	850 (2.5)	<0.0001

Chronic lung disease	499 (34.8)	10 419 (21.5)	7166 (21.5)	<0.0001

Sleep apnea	44 (3.1)	2743 (5.7)	6738 (20.2)	<0.0001

Patient diagnostic data				

LVEF, mean (SD), %	27.4 (13.0)	28.9 (12.1)	29.4 (11.9)	<0.0001

QRS duration, mean (SD), ms	115.9 (30.0)	119.4 (30.2)	119.5 (30.0)	<0.0001

QRS morphology				

LBBB	371 (25.9)	12 142 (25.0)	8088 (24.3)	0.029

RBBB	137 (9.6)	5198 (10.7)	3261 (9.8)	<0.0001

LAFB	72 (5.0)	2525 (5.2)	1578 (4.7)	0.010

LPFB	10 (0.7)	424 (0.9)	268 (0.8)	0.469

Nonspecific intraventricular conduction delay	110 (7.7)	5080 (10.5)	3723 (11.2)	<0.0001

BUN level, mean (SD), mg/dL	22.4 (13.0)	22.9 (12.9)	22.9 (12.9)	0.297

Creatinine, mean (SD), mg/dL	1.2 (1.1)	1.3 (1.2)	1.3 (1.1)	<0.0001

Systolic blood pressure, mean (SD), mm Hg	126.5 (24.1)	130.5 (22.8)	132.8 (22.4)	<0.0001

Diastolic blood pressure, mean (SD), mm Hg	71.1 (14.1)	74.0 (13.5)	75.9 (14.0)	<0.0001

ICD type				0.069

ICD	973 (67.9)	32 784 (67.5)	22 753 (68.2)	

CRT-D	461 (32.1)	15 633 (32.2)	10 514 (31.5)	

Values are reported as number and proportion with condition (%) unless indicated otherwise; continuous variables are reported as mean (SD). ICD indicates implantable cardioverter-defibrillators; BMI, body mass index; BUN, blood urea nitrogen; CABG, coronary artery bypass grafting; LAFB, left anterior fascicular block; LBBB, left bundle branch block; LPFB, left posterior fascicular block; LVEF, left ventricular ejection fraction; NYHA, New York Heart Association; PCI, percutaneous coronary intervention; RBBB, right bundle branch block.

**Table 2.** Differences in Baseline Characteristics of Included ICD Recipients With Available BMI Versus Excluded Recipients Due to Missing or Inaccurate BMI

Characteristic	Available BMI (n=83 312)	Missing or Inaccurate BMI (n=1186)	*P*
Patient demographic characteristics			

Age, mean (SD), y	62.6 (13.9)	59.6 (14.7)	<0.0001

Male sex	40 747 (72.9)	578 (70.1)	0.073

Race			

White	44 701 (80.0)	622 (75.5)	0.001

Black	9396 (16.8)	168 (20.4)	0.007

Asian	964 (1.7)	14 (1.7)	0.954

Other	803 (1.4)	20 (2.4)	0.018

Hispanic ethnicity	3300 (5.9)	71 (8.6)	0.001

Insurance payer			

Private	33 332 (59.7)	427 (51.8)	<0.0001

Medicare	28 841 (51.6)	387 (47.0)	0.023

Medicaid	7605 (13.6)	170 (20.6)	<0.0001

State specific (non-Medicaid)	1054 (1.9)	16 (1.9)	0.993

Other or self-pay	4430 (7.9)	65 (7.9)	0.965

Patient history and risk factors			

Congestive heart failure	38 080 (68.2)	582 (70.6)	0.131

NYHA class			

I	12 209 (21.9)	173 (21.0)	<0.0001

II	25 409 (45.5)	319 (38.7)	

III	17 030 (30.5)	297 (36.0)	

IV	1216 (2.2)	35 (4.2)	

Nonischemic dilated cardiomyopathy	17 145 (30.7)	299 (36.3)	0.001

Ischemic heart disease	34 317 (61.4)	450 (54.6)	<0.0001

Previous myocardial infarction	28 965 (51.8)	384 (46.6)	0.003

Previous CABG	15 651 (28.0)	200 (24.3)	0.017

Previous PCI	19 011 (34.0)	237 (28.8)	0.002

Syncope	11 007 (19.7)	154 (18.7)	0.468

Ventricular tachycardia	20 945 (37.5)	356 (43.2)	0.001

Cardiac arrest	8058 (14.4)	137 (16.6)	0.074

Atrial fibrillation/atrial flutter	14 507 (26.0)	192 (23.3)	0.083

Hypertension	43 280 (77.5)	628 (76.2)	0.390

Diabetes	20 527 (36.7)	343 (41.6)	0.004

Cerebrovascular disease	8105 (14.5)	92 (11.2)	0.007

End-stage renal disease	1892 (3.4)	19 (2.3)	0.088

Chronic lung disease	11 514 (20.6)	160 (19.4)	0.400

Sleep apnea	6087 (10.9)	141 (17.1)	<0.0001

Patient diagnostic data			

LVEF, mean (SD), %	31.3 (13.2)	30.7 (12.9)	0.237

QRS duration, mean (SD), ms	106.6 (22.9)	107.1 (24.3)	0.622

QRS morphology			

LBBB	4102 (7.3)	54 (6.6)	0.388

RBBB	4834 (8.7)	62 (7.5)	0.252

LAFB	2718 (4.9)	22 (2.7)	0.004

LPFB	374 (0.7)	7 (0.8)	0.530

Nonspecific intraventricular conduction delay	5431 (9.7)	106 (12.9)	0.003

BUN level, mean (SD), mg/dL	21.7 (12.3)	21.7 (11.9)	0.952

Creatinine, mean (SD), mg/dL	1.3 (1.2)	1.2 (0.9)	0.073

Systolic blood pressure, mean (SD), mm Hg	131.5 (22.8)	130.9 (22.1)	0.463

Diastolic blood pressure, mean (SD), mm Hg	75.2 (13.8)	75.1 (13.0)	0.721

Values are reported as number and proportion with condition (%) unless indicated otherwise; continuous variables are reported as mean (SD). ICD indicates implantable cardioverter-defibrillators; BMI, body mass index; BUN, blood urea nitrogen; CABG, coronary artery bypass grafting; LAFB, left anterior fascicular block; LBBB, left bundle branch block; LPFB, left posterior fascicular block; LVEF, left ventricular ejection fraction; NYHA, New York Heart Association; PCI, percutaneous coronary intervention; RBBB, right bundle branch block.

### Association of BMI and Procedural Complications

The risk of any complication and major complications were significantly different across BMI categories (Table 3). The crude risk of any complication and any major complication was highest in underweight patients at 5.2% and 4.3%, respectively. Underweight patients consistently had the highest crude risk of any complication among single-chamber ICD implants (3.3%), dual-chamber ICD implants (4.4%), and cardiac resynchronization therapy with defibrillator (CRT-D) implants (7.6%). The risk of individual complications varied significantly across BMI categories (Table 4). The crude risk of major complications including pneumothorax, coronary venous dissection, and device-related infection was highest in underweight patients, whereas the risk of lead dislodgement was highest in obese patients. For minor complications, the crude risk of hematoma was highest in underweight patients. Overall, in underweight ICD recipients, pneumothorax and hematoma were the 2 most common complications, occurring in 2.09% and 0.98% of implants, respectively.

**Table 3.** Risk of In-Hospital Complications, Length of Stay, and Mortality Stratified by BMI and Device Type

Type of Device and Adverse Event	Underweight, BMI ≤18.5 kg/m^2^ (n=1434)	Normal Weight, 18.5 kg/m^2^<BMI<30 kg/m^2^ (n=48 539)	Obese, BMI ≥30 kg/m^2^ (n=33 339)	*P*
All ICD types				

Any complication	74 (5.2)	1100 (2.3)	703 (2.1)	<0.0001

Major complication	61 (4.3)	899 (1.9)	614 (1.8)	<0.0001

Length of stay, median (range), d	1.0 (0 to 175)	1.0 (0 to 140)	1.0 (0 to 131)	<0.0001

In-hospital death	11 (0.8)	166 (0.3)	114 (0.3)	0.026

Single-chamber ICD	(n=418)	(n=11 467)	(n=8231)	

Any complication	14 (3.3)	131 (1.1)	109 (1.3)	0.0003

Major complication	11 (2.6)	101 (0.9)	92 (1.1)	0.0011

Length of stay, median (range), d	1.0 (0 to 175)	1.0 (0 to 88)	1.0 (0 to 131)	<0.0001

In-hospital death	1 (0.2)	28 (0.2)	19 (0.2)	0.982

Dual-chamber ICD	(n=541)	(n=20 944)	(n=14 263)	

Any complication	24 (4.4)	408 (1.9)	237 (1.7)	<0.0001

Major complication	20 (3.7)	325 (1.6)	202 (1.4)	<0.0001

Length of stay, median (range), d	1.0 (0 to 94)	1.0 (0 to 84)	1.0 (0 to 94)	<0.0001

In-hospital death	3 (0.6)	63 (0.3)	43 (0.3)	0.570

Cardiac Resynchronization Therapy ICD	(n=475)	(n=16 128)	(n=10 845)	

Any complication	36 (7.6)	561 (3.5)	357 (3.3)	<0.0001

Major complication	30 (6.3)	473 (2.9)	320 (3.0)	<0.0001

Length of stay, median (range), d	1.0 (0 to 29)	1.0 (0 to 140)	1.0 (0 to 90)	<0.0001

In-hospital death	7 (1.5)	75 (0.5)	52 (0.5)	0.008

Values are reported as number and proportion with condition (%) unless indicated otherwise. ICD indicates implantable cardioverter-defibrillators; BMI, body mass index.

**Table 4.** Individual Complications Stratified by BMI

All Complications	Underweight, BMI ≤18.5 kg/m^2^ (n=1434)	Normal Weight, 18.5 kg/m^2^<BMI<30 kg/m^2^ (n=48 539)	Obese, BMI ≥30 kg/m^2^ (n=33 339)	*P*
Major complications				

Lead dislodgement	11 (0.77)	389 (0.80)	373 (1.12)	<0.0001

Pneumothorax	30 (2.09)	191 (0.39)	39 (0.12)	<0.0001

Cardiac arrest	6 (0.42)	117 (0.24)	90 (0.27)	0.34

Coronary venous dissection	6 (0.42)	68 (0.14)	44 (0.13)	0.019

Pericardial tamponade	2 (0.14)	55 (0.11)	25 (0.07)	0.20

Device-related infection	5 (0.35)	38 (0.08)	27 (0.08)	0.0023

Cardiac perforation	1 (0.07)	42 (0.09)	14 (0.04)	0.057

Transient ischemic attack or stroke	0 (0.00)	23 (0.05)	16 (0.05)	0.71

Myocardial infarction	1 (0.07)	14 (0.03)	7 (0.02)	0.47

Urgent cardiac surgery	1 (0.07)	12 (0.02)	8 (0.02)	0.56

Hemothorax	0 (0.00)	15 (0.03)	3 (0.01)	0.095

Peripheral embolus	1 (0.07)	5 (0.01)	3 (0.01)	0.094

Valve injury	0 (0.00)	1 (0.00)	0 (0.00)	0.70

Minor complications				

Hematoma	14 (0.98)	140 (0.29)	63 (0.19)	<0.0001

Drug reaction	1 (0.07)	32 (0.07)	16 (0.05)	0.57

Conduction block	1 (0.07)	19 (0.04)	14 (0.04)	0.84

Set screw problem	0 (0.00)	20 (0.04)	8 (0.02)	0.33

Venous obstruction	1 (0.07)	13 (0.03)	9 (0.03)	0.63

Peripheral nerve injury	0 (0.00)	0 (0.00)	0 (0.00)	—

Values are reported as number and proportion with condition (%). BMI indicates body mass index.

Because those with chronic lung disease were found to experience a pneumothorax more often than those without chronic lung disease (0.41% versus 0.28%; *P*=0.005) and because such patients may more likely be underweight, we performed a separate analysis to determine if our findings related to the risk of pneumothorax were confounded by the presence of chronic lung disease: underweight status continued to be associated with a greater odds of pneumothorax after adjustment for chronic lung disease only (OR, 5.18; 95% CI, 3.50 to 7.67; *P*<0.0001) and after adjustment for all covariates including chronic lung disease (OR, 4.19; 95% CI, 2.80 to 6.26; *P*<0.0001). Obese ICD recipients had a lower odds of pneumothorax compared with normal weight recipients after multivariable adjustment (OR, 0.36; 95% CI 0.25 to 0.52; *P*<0.0001).

**Figure 1.**
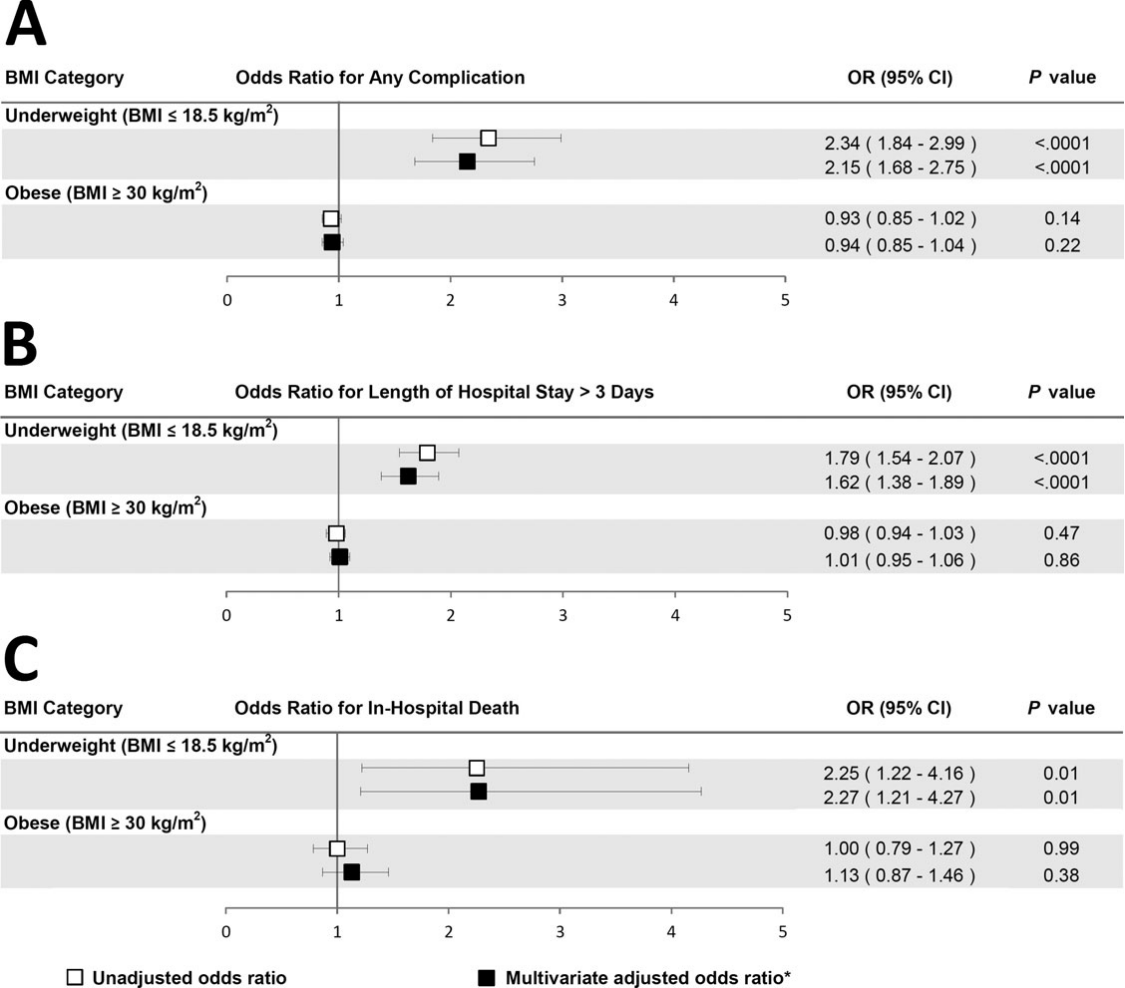
Unadjusted and adjusted ORs of clinical outcomes including any complication (A), length of hospital stay >3 days (B), and in-hospital mortality (C) among underweight and obese patients undergoing ICD) placement are depicted. The reference group for all analyses are normal weight patients (18.5 kg/m^2^<BMI<30 kg/m^2^). ICD indicates implantable cardioverter-defibrillators; BMI, body mass index. *Adjusted for patient demographics (age, sex, race), comorbidities (congestive heart failure, New York Heart Association class, syncope, ventricular tachycardia, atrial fibrillation, nonischemic cardiomyopathy, ischemic heart disease, previous myocardial infarction, previous coronary artery bypass graft surgery, previous percutaneous coronary intervention, cerebrovascular disease, chronic lung disease, diabetes, sleep apnea, hypertension, end-stage renal disease, cardiac arrest), and diagnostic information (left ventricular ejection fraction, QRS duration, ECG conduction abnormality, blood urea nitrogen level, serum creatinine).

Overall, compared with those with normal weight, underweight patients had a significantly greater odds of a procedural complication in both unadjusted and multivariable adjusted analyses (Figure 1A). Obese patients had similar odds of any complication as normal weight patients. When ICD recipients were dichotomized into underweight versus the remainder of the cohort (normal weight and obese combined), a greater odds of any complication in underweight patients was still observed (adjusted OR, 2.19; 95% CI, 1.72 to 2.80; *P*<0.0001). These findings were consistent across device types: compared with normal weight ICD recipients, underweight ICD recipients had greater odds of any complication from the implantation of a single-chamber device (OR, 1.61; 95% CI, 1.38 to 1.89; *P*<0.0001), a dual-chamber device (OR, 2.23; 95% CI 1.45 to 3.42; *P*=0.0003), and a CRT-D device (OR, 2.05; 95% CI 1.44 to 2.94; *P*<0.0001). Conversely, compared with normal weight ICD recipients, obese ICD recipients had similar odds of any complication from the implantation of a single-chamber device (OR, 1.00; 95% CI, 0.95 to 1.06; *P*=0.884), a dual-chamber device (OR, 0.85; 95% CI, 0.72 to 1.01; *P*=0.067), and a CRT-D device (OR, 0.95; 95% CI, 0.82 to 1.10; *P*=0.515). Compared with those with BMI data, those with missing or inaccurate BMI measurements did not exhibit a difference in any complication (OR, 1.07; 95% CI, 0.70 to 1.64; *P*=0.757).

### Association of BMI and Length of Hospital Stay

**Figure 2.**
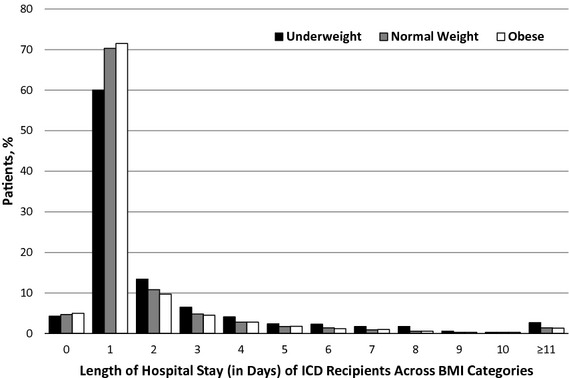
Distribution of length of hospital stay from implant to discharge in underweight, normal weight, and obese ICD recipients. ICD indicates implantable cardioverter-defibrillators; BMI, body mass index.

The median length of hospital stay was 1 day, and the total length of hospital stay was ≤3 days in 90% of patients. Although the median length of hospital stay was similar among underweight, normal weight, and obese ICD recipients, the largest upper bound of the interquartile ranges was observed in underweight patients (Table 3). A lower proportion of underweight patients stayed ≤1 days (Figure 2).

In both unadjusted and multivariable adjusted analyses, underweight patients had an increased odds of a hospital stay >3 days, whether compared with normal weight patients (Figure 1B) or compared with everyone else in the cohort (OR, 1.61; 95% CI, 1.38 to 1.89; *P*<0.0001). Obese patients in both unadjusted and multivariable adjusted analyses had a similar odds of a hospital stay >3 days compared with normal weight patients. Patients with available BMI measurements had a decreased odds of hospital stay >3 days compared with excluded patients with missing or inaccurate BMI data (OR, 0.75; 95% CI 0.62 to 0.91; *P*=0.004).

### Association of BMI and In-Hospital Death

The crude risk of in-hospital death was highest in underweight ICD recipients at 0.8% (Table 3). Of all ICD types, underweight CRT-D recipients had the highest crude risk of in-hospital mortality at 1.5%. However, in adjusted analysis, there was no overall significant interaction between BMI category and device type in the risk of in-hospital death (*P*=0.79 for interaction).

Underweight ICD recipients had a greater odds of in-hospital death compared with normal weight ICD recipients in unadjusted and multivariable adjusted analyses, whether compared with normal weight patients (Figure 1C) or the remainder of the cohort (OR, 2.19; 95% CI, 1.18 to 4.09; *P*=0.014). Obese patients had similar unadjusted and multivariable adjusted odds of in-hospital death as normal weight patients. There was no difference in the adjusted odds of in-hospital death in patients with available BMI measurements compared with excluded patients with missing or inaccurate BMI data (OR, 0.76; 95% CI, 0.33 to 1.75; *P*=0.518).

Overall, because female sex was associated with low BMI, we considered the possibility that sex might modify the association of low BMI with clinical outcomes. Alternative models that included BMI, sex, and the 2-way multiplicative interaction term were constructed for each clinical outcome studied. We found no evidence that sex meaningfully modified the association of BMI with in-hospital complications (*P*=0.31 for interaction), length of hospital stay >3 days (*P*=0.91 for interaction), or mortality (*P*=0.38 for interaction). Additionally, because defibrillation testing was differentially performed across BMI categories, we repeated analysis of each outcome after adjusting for induction for defibrillation testing. There were no meaningful changes to our point estimates or results when these additional analyses were performed.

## Discussion

In a large, national sample of first-time ICD recipients, we demonstrated that underweight patients had a greater risk of adverse events compared with normal weight or obese patients. Underweight patients experienced a ≍2-fold increase in the odds of in-hospital complications, length of hospital stay >3 days, and in-hospital death compared with normal weight patients in both crude and multivariable adjusted analyses. In contrast, obese patients did not exhibit an increased risk of any of these adverse events.

Previous studies examining the association of BMI with cardiac device implantation complications were small and limited to pacemaker implants.^13,14^ In a study by van Eck et al,^13^ low BMI was 1 of 6 variables associated with in-hospital adverse events in 1198 patients undergoing first-time pacemaker implantation, with an overall 10.1% incidence of adverse events. In a similar Dutch population, Udo et al^14^ found lower patient BMI to be one of several patient and device characteristics associated with pacemaker complications at 2-month and longer-term (mean of 5.8 years) follow-up. To our knowledge, this is the first study to specifically examine the association of BMI with in-hospital outcomes in ICD recipients, and it expands on previous studies of pacemaker recipients. In our study, the increased odds of adverse events in underweight ICD recipients was consistent across all 3 outcomes studied and persisted despite adjustment for patient comorbidities and clustering by hospitals. Although the absolute difference in complication and mortality risk between underweight versus normal weight patients was modest (5.2% versus 2.3% and 0.8% versus 0.3%, respectively), the relative differences were quite large. Because >80, 000 new ICD implantations occurred during the 1.5-year study period and because each outcome studied represented a major adverse event, the relative outcome differences have important ramifications relevant to patients and practicing physicians. Complications and increased hospital stays likely adversely affect patient quality of life and almost certainly translate into increased healthcare utilization and costs. Additionally, knowledge that underweight status, but not obesity, is a risk factor for adverse events and death from ICD implantation may help implanters counsel patients about procedural risks.

The mechanisms responsible for the increased risk among underweight ICD recipients are not entirely clear, but the specific complications most frequently observed have biological plausibility. Underweight patients experienced the highest risk of pneumothorax and hematoma. Pneumothorax results from the introduction of air into the pleural space, and because underweight patients have less tissue to traverse during venous access attempts for ICD lead implantation, it is plausible that a thinner body habitus may increase the risk of this complication. A pneumothorax may progress to a life-threatening condition, such as tension pneumothorax, and may require oxygen therapy or chest tube placement for treatment. The association between underweight and pneumothorax did not appear to be confounded by the presence of lung disease. The fact that obese patients exhibited significantly fewer pneumothoraces suggests that a greater amount of tissue between the target vessel and pleural space may be protective.

A device pocket hematoma results from blood collection within the generator pocket and, due to patient discomfort or concerns for possible device-related infection, may require reoperation for evacuation.^15,16^ It is plausible that underweight patients have less tissue surrounding the pocket, reducing the ability to tamponade a growing blood collection. However, it is also plausible that the higher risk of hematoma found in the underweight group may be explained by detection bias, because a thinner body habitus and less overall tissue at the device site may result in easier recognition of a hematoma. We also cannot exclude the possibility that the observed differences in adverse events were due to unmeasured characteristics such as frailty, malnourishment, and/or cancer among the underweight. However, although the presence of such confounders might more thoroughly explain the mechanism, they would still not negate the association between underweight status and adverse events. Because all adverse events in our study occurred in-hospital and therefore were relatively immediate, the end result of chronic illness per se does not sufficiently explain our results. Previous studies evaluating patients undergoing open heart surgery with coronary artery bypass grafting or valvular surgery requiring sternotomy have supported an association between low BMI and complications.^6–8^ Despite the lower risk of ICD implantation compared with open heart surgery, the influence of low BMI on adverse outcomes in ICD recipients remains important given the increasing number of patients undergoing ICD implantation.

Overall, obese ICD recipients did not exhibit increased complications. However, when subdivided into individual complications, obese ICD recipients were at increased risk of lead dislodgement. Although the reasons for this observation are unknown, it may be that a higher body mass results in increased force of movement (particularly when transitioning from a laying position during implantation to a sitting/standing position afterward) that is more likely to dislodge recently placed leads. Alternatively, difficulty with visualization of lead position and stability during implantation due to reduced x-ray penetration may more often lead to suboptimal initial lead placement.

How might information from this study be incorporated into clinical practice? There are several clinically appropriate and feasible strategies that can be entertained. First, physicians may consider underweight status as a new risk factor in counseling potential ICD patients; similarly, it may be important to know that obese patients undergoing ICD implantation do not appear to carry an increased risk. To minimize the risk of a pneumothorax, implanting physicians may consider pursuing axillary or cephalic rather than subclavian access in underweight patients. To minimize the risk of hematoma, physicians may more often consider prophylactic pressure dressings and, in select cases, a less aggressive anticoagulation strategy in underweight patients with borderline indications for anticoagulation bridging.

Our study has several limitations. First, our analyses were limited to in-hospital events during the index hospitalization. Therefore, it is possible that the risk of complications changes over longer follow-up. However, previous studies have shown that the majority of complications from ICD implantation are recognized before discharge.^17^ Indeed, complications so proximal in time to device implant are more likely to reflect those most specific to the procedure itself. Second, underweight patients comprised only 1.7% of the cohort. However, this percentage included >1400 patients, likely providing ample power to perform the analyses described. In addition, a lack of power should not result in spurious false-positive results. Third, hospitals participating in the ICD Registry are only required to submit data regarding Medicare patients receiving a primary prevention device, leaving the potential for selection bias in the reporting of patient data. To avoid this bias, we only analyzed data from hospitals that submit data from all ICD implantations. Although this may reduce the generalizability of our results, we believe it strengthens the overall validity of our findings. Selection bias may have been introduced due to unavoidable exclusion of patients with missing or inaccurate measures of BMI. However, because missing or inaccurate BMI was only significantly associated with one of the outcomes (length of stay >3 days), it is unlikely our findings were consequently systematically influenced in favor of an association between underweight status and all 3 adverse outcomes. Fourth, as with any observational study, we cannot exclude the possibility that residual confounding explains our results. However, our extensive multivariable adjustment did not meaningfully change any of our results, and it appears unlikely that an unmeasured confounder could explain a doubling of major adverse events. Fifth, although it would appear on the basis of our data that the complications of ICD implantation, led by pneumothorax and hematoma, resulted in greater hospital stays and more deaths, the nature of this registry data does not allow for a thorough investigation into mechanisms or causal pathways. Misclassification of BMI in patients with heart failure, who are prone to fluid retention, would be a concern for any study examining an association of BMI and clinical outcomes. Because body weight is a component of BMI, miscategorization of weight could introduce bias. However, any misclassification would be expected to incorrectly assign underweight (low BMI) patients to the normal weight category, which would bias results toward the null (leading to false-negative rather than false-positive findings). A one-time measurement of BMI may not accurately represent an ICD recipient's true BMI due to fluctuations in fluid status and may have introduced error in the reported associations between BMI and adverse outcomes. Similarly, data submitted to the ICD registry by implanting institutions are self-reported and therefore could be subject to bias due to underreporting. However, underreporting of adverse events would be expected to be nondifferential with respect to patient BMI, resulting in bias results toward the null. To the contrary, we found statistically significant associations between underweight status and adverse events.

In conclusion, in a large, national registry study of first-time ICD recipients (excluding patients with a previous pacemaker or ICD), underweight patients experienced a significantly greater odds of in-hospital complications, prolonged hospital stay, and in-hospital death. Obese ICD recipients did not experience significant differences in adverse outcomes compared with normal weight patients. These findings may help to inform physicians and patients concerning the risks of ICD implantation in patients of different body size and highlight specific complications that future efforts can target to mitigate risks in underweight patients.
